# Genetic Variability of the Glucose-Dependent Insulinotropic Peptide Gene Is Involved in the Premature Coronary Artery Disease in a Chinese Population with Type 2 Diabetes

**DOI:** 10.1155/2018/6820294

**Published:** 2018-03-25

**Authors:** Xiaowei Ma, Jia Huang, Difei Lu, Nan Gu, Ran Lu, Jianwei Zhang, Hong Zhang, Jianping Li, Junqing Zhang, Xiaohui Guo

**Affiliations:** ^1^Department of Endocrinology, Peking University First Hospital, Beijing, China; ^2^Department of Endocrinology, Peking University Third Hospital, Beijing, China; ^3^Department of Cardiology, Peking University First Hospital, Beijing, China

## Abstract

**Background:**

Glucose-dependent insulinotropic polypeptide (GIP) is closely related to diabetes and obesity, both of which are confirmed to increase the risk of coronary artery disease (CAD). Our study aimed to investigate whether the polymorphisms in GIP genes could affect the risk of cardiovascular disease in type 2 diabetic patients in the Chinese Han population.

**Methods:**

We selected and genotyped two haplotype-tagging single nucleotide polymorphisms (tag-SNPs) (rs2291725 C>T, rs8078510 G>A) of GIP gene based on CHB data in HapMap Phase II database (*r*^2^ < 0.8). The case-control study of Chinese Han population involved 390 diabetic patients with CAD as positive group and 276 diabetic patients without CAD as control group. Allele and genotype frequencies were compared between the two groups.

**Results:**

In dominant inheritance model, the carriers of T/T or T/C had a lower risk of CAD (OR = 0.635, 95% CI = 0.463–0.872, *p* = 0.005), even after adjustment other CAD risk factors (gender, age, BMI, smoking status, dyslipidemia, hypertension history, and diabetic duration) (OR′ = 0.769, 95% CI′ = 0.626–0.945, *p*′ = 0.013). The allele A at rs8078510 was associated with decreased risk of CAD (OR = 0.732, *p* = 0.039). *p* = 0.018 in subgroup analysis, individuals with higher BMI (≥24 kg/m^2^) had increased risk for CAD when carrying C/C at rs2291725 (OR′ = 1.291, 95% CI′ = 1.017–1.639, *p*′ = 0.036). In age < 55 men and age < 65 women, the carriers of allele C at rs2291725 had a higher risk of CAD than noncarriers (OR = 1.627, *p* = 0.015). Carriers of allele G in rs8078510 had higher susceptibility to CAD (OR = 2.049, 95% = CI 1.213–3.463, *p* = 0.007). *p* = 0.004; in addition, allele G in rs8078510 would bring higher CAD risk to the carriers who ever smoked (OR = 1.695, 95% CI = 1.080–2.660, *p* = 0.021).

**Conclusion:**

The genetic variability of GIP gene is associated with CAD and it may play a role in the premature CAD in the Chinese Han population with type 2 diabetes.

## 1. Introduction

Glucose-dependent insulinotropic polypeptide (GIP), as an incretin, is synthesized and secreted by K cells of duodenum and jejunum, mainly after feeding, and then rapidly degradated by circular DPP-4 (dipeptidyl peptidase-4). GIP has the glucose-dependent insulinotropic effect by interacting with its receptor GIPR. It can also help increase the transcription of insulin gene and the expression of GLUT-1 (glucose transporter-1) and hexokinase-1 genes [1]. Besides, GIP improves the proliferation of pancreatic *β* cells and prevents them from apoptosis [2–8]. At the level of fasting blood glucose (about 5.1 mmol/L), GIP can promote the secretion of pancreatic glucagon and weaken the insulinotropic effect refraining from hypoglycemia. When the blood glucose reaches 6-7 mmol/L, it has the opposite effect [9, 10].

In type 2 diabetic (T2D) patients, the GIP levels in blood are higher than normal people, but its insulinotropic effect is attenuated, which is called GIP resistance [11]. The GIP resistance impairs the glucose regulation and the stimulating effect on adipogenesis, which contributes to insulin resistance. GIP can also directly protect vessels from atherosclerosis. Nagashima et al. [12] infused the *Apoe^−/−^* mice with GIP_1–42_ for 4 weeks and found that the plaques in the aorta in *Apoe^−/−^* mice were obviously smaller than the control group. Interestingly, some of the scientists found that GIP could decrease the mRNA expressions of VCAM-1 (vascular cell adhesion molecule-1), ICAM-1 (intercellular cell adhesion molecule-1), and PAI-1 (plasminogen activator inhibitor-1) and suppress the formation of foam cells.

GIP plays an important role in the regulation of glucose and lipid metabolism and protects vessels from atherosclerosis. The GIP-encoding gene located in ch17q21.3-q22 spans 10 kb containing 6 exons. The genetic variations in GIP gene may be predisposing risk factors for CAD especially in T2D patients through its gene expressions and/or molecular function impairment.

## 2. Materials and Methods

### 2.1. Subjects

The method to select enrolled patients was described by Wei et al. [13]. A total of 666 unrelated Chinese Han subjects with T2D were selected, among which 390 were CAD positive and 276 were CAD negative. At Peking University First Hospital and in this research as well, CAD positive indicates a stenosis ≥ 50% in at least one of the major coronary arteries or their main branches, while CAD negative represents the coronary stenosis of any main coronary arteries and main branches were <50%. The CAD results were confirmed by cardiac angiography or high specific spiral computer tomography (CT) scanning of coronary arteries.

Other known cardiovascular risk factors and demography data of all subjects were collected based on their medical records, including gender, age, body mass index (BMI), fasting plasma glucose (FPG), the history of dyslipidemia, hypertension (blood pressure ≥ 140/90 mmHg or receiving any antihypertensive medications), and smoking status.

### 2.2. Single Nucleotide Polymorphism Genotyping

Genomic DNA of each sample was extracted from peripheral blood leukocytes using salting out procedure (Bioteke Whole Blood DNA Extraction Kit), as the methods described in Wei et al. [13].

Six exons were contained in GIP in ch17q21.3-q22. The total 2 haplotype-tagging SNPs at GIP locus from CHB data in HapMap Phase II database (http://www.hapmap.org) (R#27, *r*^2^ < 0.8, MAF ≥ 0.05) were selected, including rs2291725 (C>T) and rs8078510 (G>A). The 2 SNPs were genotyped using polymerase chain reaction-restriction fragment length polymorphism (PCR-RFLP), and the genotyping success rates were 95%–100%. Direct DNA sequencing was applied to further confirm the genotypes for each SNP for 5% of the cases and controls with the concordance rates between RFLP and DNA sequencing of 100%.

### 2.3. Statistical Analysis

Both clinical and laboratorial data were reported by mean ± SD or percentage. Hardy-Weinberg equilibrium tests were carried out at each polymorphic locus to examine genotype distributions. Based on Haploview 4.2 (haplotypes are estimated using an accelerated EM algorithm), linkage disequilibrium (LD) and haplotype analysis were also conducted for all samples.

The SPSS statistical package (SPSS version 14.0, USA) was applied to perform statistical analyses of association of SNPs with the risk of CAD. Qualitative variables were compared via the *χ*^2^ test, while quantitative variables were compared via *t*-test on each independent sample or Mann–Whitney *U* test. Multiple logistic regression analysis was conducted to study the correlation between CAD and genotypes, with necessary adjustment for other confounders of CAD like age, gender, BMI, and smoking status. As for the association between genotypes and outcomes, a descriptive measure was applied where *p* < 0.05 was considered as statistical significance and odds ratios (ORs) were calculated with 95% confidence intervals (CIs).

## 3. Results

The demographical data between CAD-positive and CAD-negative groups were shown different in age, sex, smoking status, and lipid profile (Table 1). Hardy-Weinberg equilibrium tests for the 2 tag-SNPs are summarized in Table 2. Genotype distributions among the study population were in agreement with Hardy-Weinberg equilibrium at the 2 loci.

### 3.1. Polymorphism Distributions and Association Study

We analyzed the distribution of the 2 tag-SNPs in GIP gene in the study population. The allele A at rs8078510 was associated with decreased risk of CAD (OR = 0.732, 95% CI = 0.544–0.985, *p* = 0.039) (Table 3). In additive inheritance model, the carriers of T/C in rs2291725 had a lower risk of CAD than the carriers of C/C (OR = 0.592, 95% CI = 0.424–0.826, *p* = 0.002), even after adjustment for the known CAD risk factors (gender, age, BMI, smoking status, dyslipidemia, hypertension history, and diabetic duration) (OR′ = 0.603, 95% CI′ = 0.392–0.930, *p*′ = 0.022). As for rs8078510, the carriers of G/A had a lower risk of CAD than the carriers of G/G (OR = 0.660, 95% CI = 0.468–0.932, *p* = 0.018), even after adjustment for of the known CAD risk factors (OR′ = 0.567, 95% CI′ = 0.364–0.883, *p*′ = 0.012) (Table 4).

In dominant inheritance model, the carriers of T/T or T/C at rs2291725 had a lower risk of CAD (OR = 0.635, 95% CI = 0.463–0.872, *p* = 0.005), even after adjustment for the known CAD risk factors (OR′ = 0.769, 95% CI′ = 0.626–0.945, *p*′ = 0.013), while the carriers of G/A or A/A at rs8078510 had a lower risk of CAD compared to those of G/G (OR = 0.671, 95% CI = 0.479–0.940, *p* = 0.020; OR′ = 0.762, 95% CI′ = 0.612–0.949, *p*′ = 0.015 after adjustment) (Table 5).

### 3.2. Haplotype Analysis

Haplotypes were constructed using rs2291725 and rs8078510 depending on the physical position and the value of D′ (*D*′ > 0.5) between the 2 SNPs in one block. Common haplotypes constructed by the 2 SNPs were CG, TG, TA, and CA (Table 6). And the haplotypes CG and CA showed a significant association with CAD, the carriers of CG had a higher risk of CAD compared to noncarriers (OR = 1.381, 95% CI = 1.100–1.733, *p* = 0.005), while the carriers of CA had a lower risk of CAD compared to noncarriers (OR = 0.173, 95% CI = 0.058–0.521, *p* < 0.001).

### 3.3. BMI Subgroup Analysis

The genotype data were further analyzed after the subjects stratified by BMI. The individuals with the BMI of 24 kg/m^2^ or above had an increased risk for CAD when they carried C/C at rs2291725 (OR′ = 1.291, 95% CI′ = 1.017–1.639, *p*′ = 0.036) (Table 7).

### 3.4. Age Subgroup Analysis

The men aged <55 and women aged <65, while carrying allele C at rs2291725 had a higher risk of CAD than noncarriers (OR = 1.627, 95% CI = 1.097–2.415, *p* = 0.015). Besides, out of the relatively young subjects, those carrying allele G at rs8078510 had a higher risk for CAD (OR = 2.049, 95% CI = 1.213–3.463, *p* = 0.007), so did the carriers of G/G than A/A or A/G (OR = 2.358, 95% CI = 1.304–4.266, *p* = 0.004; OR′ = 1.589, 95% CI′ = 1.071–2.357, *p*′ = 0.021 after adjustment) (Tables 8 and 9).

### 3.5. Smoking Subgroup Analysis

Among the subjects who had ever smoked, the carriers of allele C at rs2291725 were more susceptible to CAD than the ones carrying allele T (OR = 1.485, 95% CI = 1.036–2.129, *p* = 0.031), and those who carried genotype C/C were also at high risk for CAD (OR = 1.803, 95% CI = 1.075–3.024, *p* = 0.025; OR′ = 1.500, 95% CI′ = 1.052–2.140, *p*′ = 0.025 after adjustment); allele G at rs8078510 was associated with CAD risk (OR = 1.695, 95% CI = 1.080–2.660, *p* = 0.021) (Tables 10 and 11).

### 3.6. Accumulative Risk Analysis

We took the risk genotype of rs2291725 (C/C) and rs8078510 (G/G) as CAD risk factors, along with other recognized CAD risk factors including age, sex (male ≥ 55 y, female ≥ 65 y), smoking, and BMI ≥ 24 kg/m^2^. After calculating the number of risk factors of each sample as accumulative risk score, we analyzed the association of accumulative risk score with CAD. Compared with T2D patients without any risk factors mentioned above, those who had 3 risk factors were at higher risk for CAD (OR = 5.000, 95% CI = 1.335–18.733, *p* = 0.009). As for patients with 4 and 5 risk factors, their CAD risk increased even further (OR = 7.800, 95% CI = 2.059–29.552, *p* = 0.001 and OR = 11.026, 95% CI = 2.634–46.146, *p* < 0.001, resp.) (Table 12).

## 4. Discussion

GIP, a member of the incretin family, secreted from intestinal epithelial K cells, regulates glucose and lipid metabolism through binding to GIP receptors. Apart from that, GIP also has direct effects on cardiovascular system protection [14]. It could be hypothesized that the polymorphisms in the GIP gene may be associated with CAD, especially in type 2 diabetic populations.

In this study, we found that, in dominant inheritance model, the carriers of T/T or T/C of rs2291725 had lower CAD risk compared to C/C carriers. At rs2291725 when allele T is replaced by allele C, there will be a missense mutation, ser103gly, which may affect the structure and function of GIP. A recent GWAS (genome-wide association study) revealed that rs46522 was a CAD risky SNP [15]. Interestingly, rs46522 was at strong linkage disequilibrium with rs2291725 in the Chinese Han population, which could be interpreted that the rs2291725 in GIP gene could affect genetic susceptibility to CAD in the population. And in overweight/obese patients, the homozygotes of CC at rs2291725 had significantly higher risk for CAD compared to noncarriers, which indicated that to control body weight would be a much more important strategy for the genetic susceptible individuals.

Rs8078510, the other undocumented SNP, was also found associated with CAD risk in type 2 diabetic patients in the Chinese Han population. Patients carrying minor allele A exhibited an almost one-third lower risk of CAD than noncarriers. Rs8078510 is located in the first intron of GIP gene, close to 5′ region, which may have an effect on the transcription and translation of genes [16]. Besides, it may be at strong linkage disequilibrium with a CAD risky gene as a genetic marker.

Furthermore, the carriers of haplotype CA of rs2291725 and rs8078510 had a four-fifth lower risk of CAD compared to noncarriers (95% CI = 0.058–0.521, *p* < 0.001). Importantly, the carriers of the haplotype CG constructed by the 2 risky alleles, respectively, at rs2291725 and rs8078510 even had a 40% higher risk of CAD even compared to the any other noncarriers (95% CI = 1.100–1.733, *p* = 0.005). Both rs2291725 and rs8078510 could be better as genetic dominants to screen individuals at high risk for CAD than only rs2291725.

The early-onset CAD is a threatening social-economic issue. In the study, we found that rs2291725 and rs8078510 in association with CAD especially played an important role in the younger patients (age < 55 in male and age < 65 in female) (OR 1.6–2.0, *p* < 0.05). It indicated that the SNPs might be used to screen the subjects at high risk for premature coronary artery disease.

GIP is related with both diabetes and obesity [17]. Recent study proved that GIP regulated directly on the metabolism in adipocytes through a naturally occurring variant of GIPR (E354Q) [18]. In our study, overweight and obesity subjects had an increased risk for CAD when they carried C/C at rs2291725 (*p*′ = 0.036), which was in accordance with previous studies.

Unexpectedly, while regarding rs2291725 and rs8078510 as genetic risk factors for CAD together with age, sex, BMI, and smoking, the more the number of risk factors that the individuals were carrying, the bigger the odds ratios were for CAD risk (*p* < 0.05). It could be interpreted clinically that it should be rather more important to screen out the subjects at high risk of CAD by the genetic factors in combination with regular factors and earlier intervene those patients on controllable risk factors.

In general, the study showed that the rs2291725 and rs8078510 could be taken as genetic risk factors for CAD along with traditional ones as sex, BMI, smoking, and so on, at least in the patients with type 2 diabetes.

There were some limitations in our study. The sample size was relatively small and the clinical features were not perfectly matched between the case and control groups, which might incur the possibility to introduce bias. Further functional and prospective studies on the GIP gene are required before *GIP* polymorphisms could be used as predictors of CAD risk in patients with type 2 diabetes in the Chinese Han population.

## Figures and Tables

**Table 1:** The phenotypic characteristics of the study population.

	CAD positive	CAD negative	*p*
	(*n* = 390)	(*n* = 276)	
Gender (M/F)	257/133	118/158	<0.001
Age (y)	64 (57–72)	61 (55–69)	0.001
T2DM history (y)	8 (3–13)	7 (3–12)	0.769
Hypertension history (%)	76.2	70.7	0.110
FPG (mmol/L)	6.45 (5.59–7.80)	6.42 (5.47–7.64)	0.263
HbA1c (%)	6.8 (6.3–7.8)	6.7 (6.1–7.5)	0.080
TG (mmol/L)	1.39 (0.96–1.93)	1.40 (0.97–2.03)	0.615
TC (mmol/L)	4.00 ± 1.04	4.24 ± 0.92	0.004
LDL-c (mmol/L)	2.26 (1.79–2.91)	2.45 (1.91–2.94)	0.071
HDL-c (mmol/L)	0.95 (0.80–1.10)	1.03 (0.86–1.23)	<0.001
BMI (kg/m^2^)	26.39 ± 3.37	26.19 ± 3.61	0.472
Smoking history (%)	51.5	34.1	<0.001

**Table 2:** The Hardy-Weinberg equilibrium test.

Gene	SNPs	*n*	^2^(*p*)
GIP	rs2291725		
	C/C	102	3.113 (0.078)
	C/T	140	
	T/T	30	
	rs8078510		
	G/G	174	2.993 (0.084)
	G/A	90	
	A/A	5	

**Table 3:** Association of allele frequencies at 2 SNPs with CAD in type 2 diabetic patients.

Gene	SNPs	CAD positive *n* (%)	CAD negative *n* (%)	OR (95% CI)	*p*
GIP	rs2291725				
	C	526 (68.3)	344 (63.2)		
	T	244 (31.7)	200 (36.8)	0.798 (0.633–1.005)	0.055
	rs8078510				
	G	658 (85.7)	438 (81.4)		
	A	110 (14.3)	100 (18.6)	0.732 (0.544–0.985)	0.039

**Table 4:** The association of SNPs with CAD risk in additive inheritance mode.

Gene	SNPs genotype	CAD positive *n* (%)	CAD negative *n* (%)	OR (95% CI)	*p*	OR′ (95% CI′)	*p*′
GIP	rs2291725						
	C/C	187 (48.6)	102 (37.5)				
	C/T	152 (39.5)	140 (51.5)	0.592 (0.424–0.826)	0.002	0.603 (0.392–0.930)	0.022
	T/T	46 (11.9)	30 (11.0)	0.836 (0.498–1.406)	0.500	0.709 (0.502–1.001)	0.051
	rs8078510						
	G/G	281 (73.2)	174 (64.7)				
	G/A	96 (25.0)	90 (33.5)	0.660 (0.468–0.932)	0.018	0.567 (0.364–0.883)	0.012
	A/A	7 (1.8)	5 (1.8)	0.867 (0.271–2.774)	0.810	1.065 (0.427–2.653)	0.893

OR′, 95% CI′, and *p*′ were calculated by logistic regression analysis after adjustment for other known CAD risk factors (age, BMI, smoking status, dyslipidemia history, hypertension history, and diabetic duration).

**Table 5:** The association of SNPs with CAD risk in dominant inheritance mode.

Gene	SNPs genotype	CAD positive *n* (%)	CAD negative *n* (%)	OR (95% CI)	*p*	OR′ (95% CI′)	*p*′
GIP	rs2291725						
	C/C	187 (48.6)	102 (37.5)				
	C/T + T/T	198 (51.4)	170 (62.5)	0.635 (0.463–0.872)	0.005	0.769 (0.626–0.945)	0.013
	rs8078510						
	G/G	281 (73.2)	174 (64.7)				
	G/A + A/A	103 (26.8)	95 (35.3)	0.671 (0.479–0.940)	0.020	0.762 (0.612–0.949)	0.015

OR′, 95% CI′, and *p*′ were calculated by logistic regression analysis after adjustment for other known CAD risk factors (age, BMI, smoking status, dyslipidemia history, hypertension history, and diabetic duration).

**Table 6:** Association of the frequencies of common haplotypes with CAD.

Haplotype (rs2291725 and rs8078510)	CAD positive (%)	CAD negative (%)	OR (95% CI)	*p*
CG	67.8	60.3	1.381 (1.100–1.733)	0.005
TG	17.8	21.1	0.810 (0.615–1.067)	0.133
TA	13.9	15.8	0.862 (0.634–1.170)	0.352
CA	0.6	2.9	0.173 (0.058–0.521)	<0.001

**Table 7:** The association of SNPs with CAD risk in overweight/obesity subgroup in dominant inheritance mode.

Gene	SNPs genotype	CAD positive *n* (%)	CAD negative *n* (%)	OR (95% CI)	*p*	OR′ (95% CI′)	*p*′
GIP	rs2291725						
	C/C	142 (47.8)	83 (40.5)	1.347 (0.939–1.930)	0.105	1.291 (1.017–1.639)	0.036
	C/T + T/T	155 (52.2)	122 (59.5)				

OR′, 95% CI′, and *p*′ were calculated by logistic regression analysis after adjustment for other known CAD risk factors (age, BMI, smoking status, dyslipidemia history, hypertension history, and diabetic duration).

**Table 8:** Allele distributions of GIP SNPs in male < 55 y and female < 65 y.

Gene	SNPs	CAD positive *n* (%)	CAD negative *n* (%)	OR (95% CI)	*p*
GIP	rs2291725				
	C	156 (72.9)	157 (62.3)	1.627 (1.097–2.415)	0.015
	T	58 (27.1)	95 (37.7)		
	rs8078510				
	G	190 (88.8)	197 (79.4)	2.049 (1.213–3.463)	0.007
	A	24 (11.2)	51 (20.6)		

**Table 9:** Genotype distributions of GIP SNPs in male < 55 y and female < 65 y in dominant inheritance mode.

Gene	SNPs genotype	CAD positive *n* (%)	CAD negative *n* (%)	OR (95% CI)	*p*	OR′ (95% CI′)	*p*′
GIP	rs2291725						
	C/C	55 (51.4)	44 (34.9)	1.971 (1.164–3.339)	0.011	1.346 (0.958–1.892)	0.087
	C/T + T/T	52 (48.6)	82 (65.1)				
	rs8078510						
	G/G	85 (79.4)	77 (62.1)	2.358 (1.304–4.266)	0.004	1.589 (1.071–2.357)	0.021
	G/A + A/A	22 (20.6)	47 (37.9)				

OR′, 95% CI′, and *p*′ were calculated by logistic regression analysis after adjustment for other known CAD risk factors (age, BMI, smoking status, dyslipidemia history, hypertension history, and diabetic duration).

**Table 10:** Allele distributions of GIP SNPs in smoking patients.

Gene	SNPs	CAD positive *n* (%)	CAD negative *n* (%)	OR (95% CI)	*p*
GIP	rs2291725				
	C	267 (67.8)	109 (58.6)	1.485 (1.036–2.129)	0.031
	T	127 (32.2)	77 (41.4)		
	rs8078510				
	G	345 (86.3)	148 (78.7)	1.695 (1.080–2.660)	0.021
	A	55 (13.8)	40 (21.3)		

**Table 11:** Genotype distributions of rs2291725 in smoking patients in dominant inheritance mode.

Gene	SNPs genotype	CAD positive *n* (%)	CAD negative *n* (%)	OR (95% CI)	*p*	OR′ (95% CI′)	*p*′
GIP	rs2291725						
	C/C	91 (46.2)	30 (32.3)	1.803 (1.075–3.024)	0.025	1.500 (1.052–2.140)	0.025
	C/T + T/T	106 (53.8)	63 (67.7)				

OR′, 95% CI′, and *p*′ were calculated by logistic regression analysis after adjustment for other known CAD risk factors (age, BMI, dyslipidemia history, hypertension history, and diabetic duration).

**Table 12:** The association of accumulative risk score with CAD risk in T2D patients.

Risk factors	CAD positive *n* (%)	CAD negative *n* (%)	OR (95% CI)	*p*
0	3 (0.7)	10 (3.6)	1	1
1	28 (7.2)	42 (15.2)	2.222 (0.561–8.798)	0.247
2	79 (20.3)	81 (29.4)	3.251 (0.862–12.254)	0.068
3	120 (30.8)	80 (29.0)	5.000 (1.335–18.733)	0.009
4	117 (30.0)	50 (18.1)	7.800 (2.059–29.552)	0.001
5	43 (11.0)	13 (4.7)	11.026 (2.634–46.146)	<0.001

Accumulated risk factors included risk genotype of rs2291725 (C/C) and rs8078510 (G/G), age, sex (male ≥ 55 y, female ≥ 65 y), smoking, and BMI ≥ 24 kg/m^2^.

## Abbreviations

GIP: Glucose-dependent insulinotropic polypeptide
DPP-4: Dipeptidyl peptidase-4
T2D: Type 2 diabetes
CAD: Coronary artery disease
CT: Computer tomography
SNPs: Single nucleotide polymorphisms
PCR: Polymerase chain reaction
RFLP: Restriction fragment length polymorphism
BMI: Body mass index
FPG: Fasting plasma glucose
LD: Linkage disequilibrium
ORs: Odds ratios
CIs: Confidence intervals
GLUT-1: Glucose transporter-1
VCAM-1: Vascular cell adhesion molecule-1
ICAM-1: Intercellular cell adhesion molecule-1
PAI-1: Plasminogen activator inhibitor-1
GWAS: Genome-wide association study.
